# Academic monitoring and support of undergraduate nursing education programme: A middle-range theory

**DOI:** 10.4102/curationis.v41i1.1881

**Published:** 2018-12-03

**Authors:** Prenola D. Mudaly, Ntombifikile G. Mtshali

**Affiliations:** 1 School of Nursing and Public Health, University of KwaZulu-Natal, South Africa

## Abstract

**Background:**

Globally, there is consensus on the need for student support to address high student attrition and low throughputs, especially in nursing and midwifery programmes.

**Objectives:**

This study analysed the implementation of academic monitoring and support (AMS) in an undergraduate nursing programme to generate a context-informed academic monitoring and support middle-range theory.

**Method:**

An ethnographic design and grounded theory approach were adopted in this study. Data sources included individual and focus group interviews, observations, reflective conversations and document analysis. Ethical clearance was obtained from the University Research Ethics Board, and ethical principles were maintained throughout the study.

**Results:**

The country’s contextual conditions emerged as conditions that necessitated a comprehensive approach to student support to increase throughput in a nursing programme that attracted students from diverse backgrounds. A shared common vision, supportive leadership, collaboration and investing resources in a student support programme that uses a comprehensive and holistic approach emerged as key to an AMS model that will yield the desired outcomes. Major concepts in an AMS middle-ranged theory generated included education for social justice, visionary leadership, comprehensive, holistic and intentional student support, AMS pillars, AMS threats and process and goal-oriented consequences.

**Conclusion:**

Academic monitoring and support is a tool used to facilitate access of all deserving students to an undergraduate nursing programme and to ensure that they all have an equal chance to succeed academically, resulting in improved throughput rates. Strengthening support in clinical settings is recommended and further research to improve effectiveness of AMS programmes is suggested.

## Introduction

Student attrition and dropout rate affects the country’s planned nursing and midwifery workforce projections (Ramkilowan & Mtshali 2015). Nursing programmes strategically draw diverse students, including those from remote and rural areas, as a way of addressing the gross shortage of nurses in such settings. The emerging evidence suggests that students recruited from rural areas, if they do well and are successful in their studies, are likely to go back and serve in their communities (Ross 2014; Ross & MacGregor 2012; World Health Organization 2013). However, drawing students from disadvantaged backgrounds has not been without challenges, as they are regarded as underprepared academically, with limited academic skills to cope with the demands of higher education (Jones et al. 2008). The Council on Higher Education (CHE) in 2013 reported an increase in failure rates and student attrition (CHE 2013). This prompted a number of questions that enquired into the effectiveness and efficiency of the support provided to the students (CHE 2013; Jones et al. 2008). Providing access to students from disadvantaged backgrounds without the required support is not an opportunity for such students, as many of them fail to complete their studies because they lack academic, social, financial and personal support (Tinto 2014). In addition, Tinto (2014) contends that improving student success is not by chance. There has to be a goal-focused, well thought-through plan that is accompanied by a coherent set of policies and resources.

The concern over high failure and student dropout rates led to the intensification of structured student support interventions aimed at widening participation that is accompanied by successful completion of studies, with the Department of Higher Education and Training (DHET) even committing to funding student support resources through a Teaching Development Grant (Department of Education 2004; Essack et al. 2010). This funding and other investments made towards student support interventions has made it possible for higher education institutions to introduce a range of student support interventions from as early as pre-enrolment up to completion of studies (Essack et al. 2010) and they are starting to yield positive results. The students drawn from disadvantaged backgrounds are reported to be coping well and succeeding in their studies (Essack et al. 2010; Ross 2014; Ross & MacGregor 2012).

## Problem statement

Institutions have reported a number of student support interventions which are producing positive outcomes (Ravanipour & Bahreini 2015; Robinson & Niemer 2010; Secomb 2008; Stone, Cooper & Cant 2013; Thalluri, O’Flaherty & Shepherd 2014). However, there are some concerns about the inconsistencies in student support practices, processes and interventions, which affect the effectiveness of the student support programmes and their impact (Ntakana 2011). Authors such as Borden, Vithal and Dhunpath (2013) argue that, whilst there is consensus on the need to support the students, there is no agreed-upon form of effective student support that will generate the desired results. Tinto (2014) asserts that although great strides have been made in the field of student support and success, more work still needs to be done in terms of generating explanatory theories guiding this field, which is characterised by a number of disagreements and confusion. A number of models and theories related to student support, engagement, integration and success have been developed; however, these are not comprehensive in that they focus on selected aspects of student support and some address student persistence and student success separately (Tinto 1975; Tinto & Pusser 2006). There is a gap in the existing scholarly work in that there is no model of student success that is comprehensive and longitudinal in nature that may be adopted by education institutions as a guide to follow in order to enhance student success (Tinto 2014). Tinto, in a presentation he made in South Africa, indicated a need to develop theories of student success that are appropriate to the South African context and that will focus on not only students completing their studies but also how academic monitoring and support (AMS) could be used as a vehicle to help students stay and finish their studies in ways that lead to powerful and meaningful learning (Tinto 2014). In 2006, the undergraduate nursing education programme of interest to this study implemented, for the first time in a structured manner, AMS. However, the introduction of AMS in an undergraduate programme was not underpinned by any clearly articulated theoretical or philosophical basis, resulting in its lack of grounding. This discrepancy was the result of pressure to come up with urgent interventions to address high failure rates (Mudaly & Mtshali 2016).

## Aim of the study

The study aimed at analysing and describing the implementation of AMS in an undergraduate nursing education programme to generate a middle-ranged AMS theory that is context informed.

## Methodology

This study adopted a classical ethnographic approach as part of data collection by key informant interviews, focus group discussions, document analysis, reflective conversations and participant observation in order to allow the researcher, as a data-collecting instrument, to be immersed in the culture of AMS in an undergraduate programme. The grounded theory data analysis framework suggested by Strauss and Corbin (1990) was used to analyse data and to generate a middle-range theory. Pettigrew (2000) supports the use of ethnography and grounded theory because ethnography brings in the richness of the culture of the key informants and the rigorous process of data analysis followed in grounded theory facilitates the emergence of a theory. The initial key informants were purposively selected and theoretical sampling was used with the guidance of the key informant. A total of 40 key informants participated in individual and focus group interviews and they included Bachelor of Nursing students (24), peer-wellness mentors (4), academic mentors or student tutors (4), nurse educators (4), the AMS coordinator, student counsellor and two academic development officers. Document analysis and naturalistic conversations with the informants and observations were used as other sources of data (Whitehead 2005). The three levels of data analysis (open, axial and selective coding) in Strauss and Corbin (1990) were followed. Open and axial coding using Strauss and Corbin’s paradigm resulted in a conceptual framework and the selective coding process resulted in a middle-range theory. The process of theory generation included constant comparative analysis of data, engaging in selective sampling of literature to determine the fit of findings to the already existing models and theories and validating the emerging model with data from key informants which, according to Strauss and Corbin (1990), completes the grounding of a theory.

## Trustworthiness

Academic rigour principles (trustworthiness) were followed as stated in Lincoln and Guba (1985). Credibility was ensured through providing a detailed and thick description of the research plan and of the data analysis process, verifying data with the key informants and having a co-coder. Dependability was ensured through triangulating data sources, consulting an expert in grounded theory, having a peer-coder and having the emerging categories checked by a research mentor. Transferability was ensured through providing a thick description of the study context, settings, procedures and findings. Confirmability was ensured by transcribing interviews verbatim, verifying transcribed data with key informants and through purposive sampling to allow the middle-range theory to emerge from the key informants who are involved in AMS.

## Ethical considerations

Gatekeeper permission by a letter of permission was sought from the Registrar of the Institution (HSS/0562/104D), the Dean and Head of the School, as well as the Head of the Nursing Department (HSS/0562/104D); Humanities and Social Sciences Committee (HSS/0562/104D); and Department of Health (14/RESH/2014). Informed consent was obtained from the participants and ethical principles were observed as was specified in the approved research protocol.

## Results

The results in this paper are part of a bigger study and they focus on the middle-range theory that emerged from analysing the culture, practices, processes and procedures during the implementation of AMS. The findings that emerged from this study were summarised into a conceptual framework (Appendix 1) that served as the basis to the middle-range theory presented in this paper. The detailed results with the conceptual framework are presented as a separate work, but are summarised in this article to lay a foundation. According to Fawcett (2005), a conceptual framework is always a precursor to a middle-range theory. The findings were organised according to Strauss and Corbin’s grounded theory analysis framework. They included antecedent, contextual and intervening conditions in implementing AMS, action and interaction strategies as well as consequences of AMS.

The results revealed that the antecedent conditions that led to the adoption of AMS in an undergraduate nursing education programme include high failure rates and students’ underpreparedness for higher education which affected their self-confidence and ability to adjust to higher education, as per the extracts below:

‘We had concerning high failure rates, especially students from disadvantaged background.’ (P1, female, 55 years old)

‘Some … tell you it is their first time using a computer, typing an assignment, (using) the internet or going into such a big library.’ P2, female, 45 years old

‘Students from rural areas struggle with … self-confidence, adjusting to university life, (and) financial management.’ (P3, female, 40 years old)

The external environment, which is DHET, Department of Health (DOH) and South African Nursing Council (SANC), in the context of this study, emerged as part of the contextual conditions that AMS responded to. AMS emerged as a tool to facilitate the process of producing graduates required by the external environment. The external environment’s transformation agenda includes widening access to all deserving students, producing graduates competent to meet the human resources needs of the country.

‘AMS is responding to DHET agenda of widening access with success.’ (P1, female, 55 years old)

‘NDOH [National Department of Health] wants us to attract and train according to the HRH [Human Resources for Health] needs of our country that is aimed at meeting the needs of all irrespective of their geographic location and financial status.’ (P1, female, 55 years old)

‘SANC … stipulates that programmes should be relevant and responsive to the country(’s) needs.’ (P1, female, 55 years old)

Data sources also revealed a number of tools that provided a basis for the implementation of AMS. These included the University Strategic Plan, which pledged to promote access and excellent services to the students to promote success; the Academic Monitoring and Exclusions policy to ensure a transparent, fair and consistent process of academic exclusion after intensive support; amendment of the amended Admission and Selection policy to use a quota system, with 15 % of the spaces reserved for students from disadvantaged schools; and the University Language policy, which allowed for bilingual teaching and learning, which was operationalised by translating nursing and midwifery terminology into isiZulu. The terminology that was translated was reported to be used in extra tutorials during supplemental instruction sessions, facilitating code switching to ensure that difficult concepts are explained in an understandable language to all members of the group, as per the extracts below:

The AMS policy commits the university to identifying under-performing students timeously and (to) providing the necessary academic support to assist students to graduate in the minimum time possible or (to) redirect them and (it) obligates students to attend and participate in the range of support that is made available. (University of KwaZulu-Natal, 2009)

‘The University’s language policy and plan (which) is intended to promote and facilitate the use of isiZulu as a language of learning, communication, instruction, and administration.’ (Participant 1, female, 55 years old)

‘Nursing terminology that was translated into isiZulu … was very helpful in extra tutorials.’ (Participant 4, female, 23 years old)

‘Admission policy amended in 2012 ensure(s) that all the programmes have at least 15% students from quintile 1 and 2 schools (so as) to open(ing) access and (to) change the university student profile.’ (Participant 1, female, 55 years old)

The action and interaction strategies in this study included a range of student support interventions, which were holistic, comprehensive, building on each other and starting from the pre-enrolment to the final year when students are prepared for the world of work. The type of support provided to the student emerged as three-pronged: academic development and support, adoption of student-centred curriculum and psychosocial support offered by student support services. The support provided was in phases: pre-enrolment, integration, engagement and transition. The pre-enrolment phase support included career counselling. Integration into the university included a compulsory orientation programme, skills-development workshops (which were compulsory at first-year level and voluntary thereafter), peer-wellness mentorship and risk profiling as part of early screening for academic risk. The engagement phase included support integrated into a student-centred transformative curriculum, academic tutoring, clinical peer-mentorship, individualised lecturer-student support and tracking and monitoring of academic performance. The students during the engagement phase had access to psychosocial support offered by the social worker from student support services. The support during the transition phase prepared the senior students for the world of work, specifically with expectations from nurses as nursing is a profession, a career option and an opportunity for further study.

‘We had to revisit our curricula in line with this policy to ensure that our curricula, as well as our teaching and assessment strategy, integrate student support.’ (P1, female, 55 years old)

‘Provide students with support as they navigate their way through their journey from 1st year through to graduation. … Interventions in our AMS (academic monitoring and support) programme are grouped into; pre-entry, integration, engagement and preparation for the world of work.’ (P3, female, 40 years old)

‘Some services offered are personal counsel(l)ing, career counselling, academic support programmes, crisis/trauma management, life skills-development programmes, peer-wellness mentoring to facilitate integration [in]to [the] university and peer-mentorship in clinical settings by senior students, which is however not formalized.’ (P5, female, 48 years old)

The intervening conditions for AMS to be successful according to this study included the institution having a common or shared vision, leadership commitment, availability of resources (funding and human resources) and student commitment to utilise provided support services. Having a shared vision is important to ensure institutional commitments to AMS implementation

‘AMS (academic monitoring and support) is integrated into our university strategy and that vision is translated down to all the levels.’ (P5, female, 48 years old)

‘[The] DVC (Deputy Vice Chancellor) Teaching and Learning at an Institutional Level sources funding to implement the AMS strategy.’ (P1, female, 55 years old)

‘Our students have to use all these services, otherwise, it is a wasted effort.’ (P5, female, 48 years old)

Data sources revealed a number of consequences associated with AMS implementation, which were grouped into process-oriented and product-oriented outcomes. The process-oriented were viewed as short-term outcomes, which included developing students’ capacity to develop self-confidence, to acquire transferable skills, to reduce anxiety and stress levels in clinical areas, to be resilient and to continue against difficult situations and to improve academic performance. The long-term outcomes included reduced dropout rates, increased pass rates and throughputs and the production of students who are change agents, and it was hoped that there would be an improved distribution of the nursing and midwifery workforce and, as a result, an improved level of health in the population in due time.

‘Most of us struggled with things (like) time management, typing assignments, writing assignments properly, and even referencing but now we are teaching the new ones because of the support we received (in our) first year.’ (P6, female, 20 years old)

‘All our modules now have pass rates of 80% and above, since we have such support through the office of the ADO [Academic Development Officer]. Since 2007 we have seen less students dropping out especially because of subjects such as anatomy and physiology.’ (P1, female, 55 years old)

‘The picture of nurses in rural and remote areas is going to change as we graduate more nurses who are from these areas. This will definitely improve the delivery of the services and health outcomes in our country.’ (P1, female, 55 years old)

The findings summarised above culminated in a conceptual framework in Appendix 1. Glaser (1992) warns that Strauss and Corbin’s grounded theory approach, which was adopted in this study, may end with full conceptual descriptions at the expense of theory development. To avoid ending up with conceptual description, which has been fitted in Strauss and Corbin’s framework, the researchers had to embark on a process that elevated conceptual descriptions to theorising. This process included engaging in a selective literature search to determine the ‘fit’ of findings from earlier studies and existing theories, the reduction of concepts and a constant comparative analysis until the core and main concepts emerged, as well as the relationship between and amongst these concepts. The emerging theory was verified against data and validated by presenting it to the selected participants for consensus reaching.

## Middle-range theory of academic monitoring and support

Middle-range theories are socially constructed (Fawcett 2005), more focused in nature, narrower than grand theories and are made of limited concepts and propositions (Fawcett 2005). This study adopted a middle-range theory in order to explain the causal relationships amongst emerging concepts of the grounded theory which in its nature gave emergence to actual experiences of informants and other sources of data collected. A middle-range theory that emerged from this study is explanatory in nature because it goes beyond identifying, describing and naming concepts as is the case with descriptive middle-range theories (Chinn & Kramer 2008; Fawcett 2005). It includes explaining causal relationships between and amongst concepts, why or how the concepts are related and the effect a change in one concept has on another concept within a theory as stated in Fawcett (2005) and Chinn and Kramer (2008). The scope of the middle-range theory in this study does not go beyond explaining relations, to predicting associations between the concepts and the direct and indirect cause and effect of relationships as is the case with predictive theories (Chinn & Kramer 2008; Fawcett 2005).

Chinn and Kramer’s (2008) six components of a theory were adopted as a guiding framework for this study. This enabled the context of the theory to be specified for application to emergent theory whilst encompassing emergent concepts towards building of the theory in aspects of its relationship built from key concepts, conceptual and theoretical that is, and assumptions of the theory.

## Goals of the academic monitoring and support middle-range theory

This middle-range theory may have multiple goals. This AMS middle-range theory aims to (1) provide a guiding framework to nursing education institutions who intend to implement a comprehensive and holistic student support programme in their undergraduate nursing education programmes, (2) provide an instrument to be used to monitor and evaluate the existing student support initiatives with the intention to enhance the nature of support provided to the undergraduate nursing students, (3) provide guidance to education programmes developing support programmes targeting students from disadvantaged backgrounds to ensure that they have a better chance of success academically and (4) contribute to the scientific body of knowledge in the area of student support with specific reference to theories guiding implementation of AMS to students from diverse backgrounds.

### Basic assumptions

Chinn and Kramer (2008) indicate that a theory is based on a number of assumptions. Assumptions represent the values and beliefs of the theory and form the basis for defining concepts (Chinn & Kramer 2008; Meleise 2007). The assumptions below form the basis of the AMS middle-range theory and are unpacked further under the definition of concepts in this theory: AMS is an instrument to facilitate change; academic and social integration into higher education is vital for student persistence and success; the institutional environment influences students’ persistence and academic success; shared vision, strategic collaborative partnerships as well as commitment by all stakeholders is central to achieving the desired AMS outcomes; and a comprehensive, holistic and intentional student support is a determinant of student success.

### Substantive concepts and conceptual relationships

**FIGURE 1:**
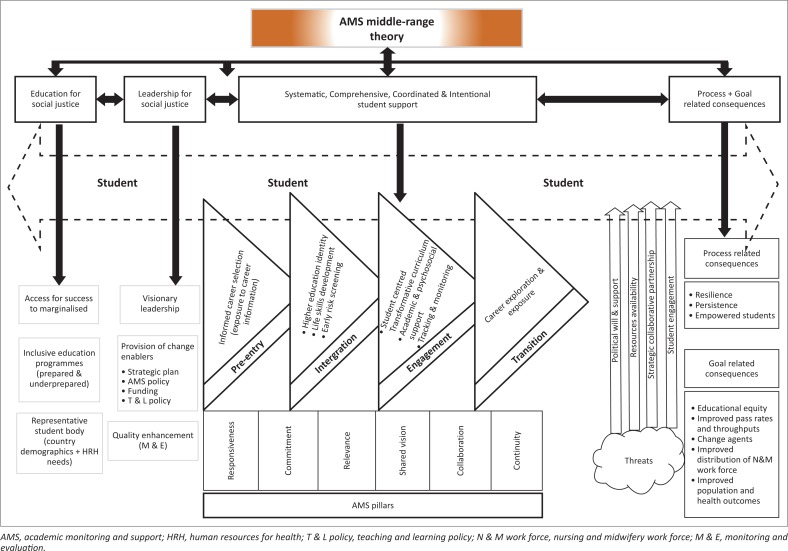
Academic monitoring and support middle-range theory.

Academic monitoring and support emerged as the major concept, as it was a phenomenon of interest in this study. The other concepts were labelled as main concepts because they contribute to the core concept. The main concepts that were directly linked to the major concept AMS include (1) education for social justice; (2) leadership for social justice; (3) systematic, comprehensive, coordinated and intentional support; (4) AMS pillars; (5) AMS threats; and (6) process- and goal-oriented consequences. The ‘student’ features as central in this theory. They were grouped according to their relationship, as in Figure 1, the AMS middle-range theory of this study. The arrows indicate relationships between and amongst the concepts. Some of the concepts are not unique to this middle-range theory but the definitions are aligned to the country’s context.

### Definition of concepts

The definition of AMS as a core concept in this theory integrates all the main concepts as they served as building blocks. Therefore, AMS is defined as a vehicle used to promote education for social justice and is facilitated by a leadership that embraces social justice principles; it is systematic, comprehensive and holistic in nature and is coordinated and intended to ensure that all students succeed irrespective of their backgrounds. AMS is underpinned by pillars such as responsiveness, commitment, relevance, shared vision, collaboration and continuity, and conditions such as political and institutional support, availability of resources, strategic collaborative partnerships and students’ utilisation of available student support initiatives may serve as threats if not well addressed. AMS has short-term and long-term consequences that lead to empowered graduates who should contribute to improving the country’s human resources needs, health service delivery and health outcomes. This definition of AMS shares some characteristics of Tinto and Pusser’s (2006:2) model of institutional support, which presents conditions of student success.

Education for social justice: The concept is defined descriptively in that AMS in this theory is conceptualised as a tool used to address social injustices of the past through making it possible to widen participation in higher education of all deserving students, irrespective of their background and their preparedness. This should result in a student body that is reflective of the country’s demographics. With access to education regarded as a human right, AMS provides a vehicle to achieve this human right. This requires amendment of admission and selection policies to create space for different categories of students who meet entry requirements, by not making them compete for spaces with students from well-resourced secondary schools. Through AMS, the discriminated and marginalised students from disadvantaged backgrounds, who are automatically excluded by the system because of their underpreparedness for higher education, have an opportunity to access higher education, and not only access it but also have an equal chance to succeed in their studies. The dehumanisation resulting from the stereotyping of students from the marginalised groups, which is related to poor academic performance and slow progression, failure and dropping out as a result of being underprepared to engage with cultural norms of higher education, is addressed through AMS.

Tinto and Pusser (2006:2) attest to the claim that some students drop out from higher education because of failure to integrate into the college or higher education culture that requires a specific set of academic and life skills in order to cope with the required discipline and specific content that this culture demands. As defined by Francis and Le Roux (2001), education for social justice is education that aims to actively address the dynamics of power domination and privilege, rooted in injustices of the past, that have resulted in socially constructed group lines, social identities and marginalisation. The distributive aspect of social justice was found to apply in this AMS theory as it is about equitable distribution of access to educational goods and to outcomes as well as to the recognition and respect for cultural difference, as stated by Cochran-Smith et al. (2009). The drive to use AMS for social justice is as a result of the external environment, DHET, NDOH and SANC, that influences nursing education.

#### Leadership for social justice

Leadership for social justice in this AMS theory is defined as leadership that is transformative in nature and that focuses on addressing and reducing marginalisation in higher education. It embraces equitable learning opportunities through inclusive education programmes and makes realities of change possible by providing students with the skills required to achieve the desired goal. This conceptualisation is in line with Shields’s (2014) view of transformative leadership in education for social justice, the goal of which is to transform both the experiences and outcomes of schooling and the inequities in the wider society. The leadership of the institution ensures this by integrating student support into the institution’s strategic plan, having a mission and vision that embraces opening access for success and student support principles and values and mobilising resources for AMS to succeed. The leadership ensures that there are enabling policies that will widen participation, ensure continuous AMS to the students and support that is integrated into the teaching and learning policies. Examples of such policies include student admission and selection policy, academic monitoring and exclusions policy, language policy and teaching, and learning and assessment policies.

#### Systematic, comprehensive, coordinated, intentional student support

This refers to the nature and type of support available to all the students irrespective of their background or academic performance. It is systematic in nature in that it is comprehensive through targeting all aspects of the student’s wellness: academic, physical (health), psychosocial (including finance), environmental (student integration into the environment) and occupational (career guidance and counselling). The students have access to a wide range of student support initiatives in line with their academic and psychosocial needs. This comprehensive support is also holistic in that the support provided to students covers all phases in higher education, from pre-entry to university to integration, engagement and transition. The aim is to provide students with constructive support as they navigate their way through their journey from first year through to graduation. The support offered during the pre-enrolment and integration phase is aimed at preventing student attrition that results from poor academic choices because of lack of information about the nursing profession and its related practical demands during work-integrated learning experiences as well as lack of academic skills required to cope with academic demands of higher education. Wells (2003) in her epidemiological approach to preventing student attrition refers to comprehensive support strategies as primary prevention strategies.

#### Pre-entry phase

This refers to support prior to the student being admitted into the higher education programme. This phase includes interventions such as counselling for academic choices to match academic ability, socio-personal-economic counselling to establish students’ ability to fund themselves in the course, seek funding, guardian and parental support for emotional and financial needs; and the need for residence for those students whose homesteads are in rural and areas far from the institution. The pre-entry phase establishes the immediate needs of the student from being enrolled into the programme.

#### Integration phase support

This refers to support provided to the student aimed at easing transition into higher education during their first academic year. This phase includes interventions such as extended pre-registration orientation, life skills development, peer-wellness mentorship and early screening for risk (risk profiling). During this phase, the students develop their own identity within higher education and they get empowered with a wide range of cognitive, psychomotor and technical skills required to cope with academic challenges in higher education, especially those students classified as underprepared for higher education. Peer-wellness mentorship refers to the psychosocial support provided to students by senior students to first-year students assisting them adjust to higher education and enhance their university experience. It also assists students to develop that sense of belonging – the possibility of connecting with the world outside their world. Being in groups as mentees takes them out of their comfort zones. They learn to engage and develop networking skills. The peer-wellness mentors are positioned to walk the students through their first-year journey. Peer-mentorship also takes place in clinical settings where first-year students are socialised into the hospital environment, which seems to be challenging to some first-year students, leading to some dropping out of the programme. Latino and Unite (2012) and Ganser and Kennedy (2012) assert that peer-wellness mentoring has significant positive effects because it enhances students’ experience in higher education and provides ongoing orientation both on campus and in residences.

Risk profiling refers to an assessment that is aimed at the early identification of those students who may be at risk of poor performance academically and who may eventually drop out of the programme. Risk profiling focuses on personal or emotional aspects, skills development, academic and clinical skills practice and on careers and health. The baseline data obtained present risk areas in each student. Potentially at-risk students who are identified early have the benefit through this early warning system to be aware of their needs and development areas. The data are used to prioritise the needs and to guide whether to offer personalised or group support, depending on the identified needs. Relevant people such as the lecturer, clinical facilitator, clinical mentors, student counsellor, skills development officer, academic tutors or supplemental instructors support the student. Those students who are found to be at risk academically are entered into a special database for monitoring and continued support. Their data are captured on the online share-point system which is a tracking system to highlight a student’s progress and to support attendance. The at-risk students are also identified through a colour-coded online student management system. Profiling first-year students for academic risk assists with early identification of actual and potential problems that may impact on the students’ academic performance.

#### Engagement phase support

This refers to a range of student support initiatives offered to the student during the stage when they are engaging in their studies as described in the conceptual framework of Appendix 1. The conceptual framework depicts how at this phase whilst the student is deeply embedded within his or her studies, the nature of support being provided includes (1) adoption of a student-centred transformative curriculum, within which student support is integrated; (2) utilising engaged learning pedagogies that embrace democratic and emancipatory learning and (3) students having access to continuous academic and psychosocial support. Student-centred transformative curricula in this theory refers to curricula that are centred on the needs and interests of the students, which utilise learning and teaching pedagogies that promote active learning and collaborative learning. Engaged learning pedagogies refer to those learning and teaching approaches that promote students’ active learning and discovery learning with students using scientific evidence to back up their arguments during the learning engagement. Examples of transformative curricula that utilised engaged learning pedagogies in this study included case-based, problem-based learning, competency-based and community-based curricula. In line with Dewey’s (2007, 1997) ideology on democracy and education, experience and education and critical theorists’ ideology such as Freire and Myra (1970) on emancipatory learning, the researcher observed during the classroom sessions that the students in the process of knowledge construction engaged with issues presented in a form of problematised situations. They had to reflect on their experiences, indicate how they applied concepts from different theories and provide evidence from research for their practice to address the presenting issue. They had to conclude by highlighting the areas where they needed to improve to manage the presenting situation better. Instead of having monologues in class, with the lecturer dominating the sessions, the students engaged in dialogue, with lecturers playing a facilitating role by asking questions that allow for in-depth exploration of the area of interest and the lecturers feature as part of the community of learners. Such classrooms are described as democratic and emancipatory as there is power-sharing with students, which allowed some autonomy. The voices of the students are more audible as they engage in dialogues with the lecturer, as they are allowed to make some choices within the scope of how to achieve the desired outcomes from the programme. Learning is enhanced through engagement and interaction between and amongst the students, as well as between the students and the lecturer (Essack 2009). The engagement phase further describes as depicted through the conceptual framework in Appendix 1 that there is a range of academic support services. These include clinical peer learning and supplemental instruction for students to actively engage with. Furthermore, the academic and psychosocial support includes tracking and monitoring of academic performance of all the students in the tests and at the end of the semester, providing students, especially those who are at risk academically, with some guidance and counselling in relation to areas where they need to improve and supporting them as they work on improving their academic performance. Building onto the support by peer learning in the integration phase is by observation of extra tutorials or supplemental instruction and in small study groups. Peer learning does not only focus on learning the subject content but also on developing life skills such as test-taking skills, note-taking, reading with comprehension and reflective practice, as students reflect on their preparation for previous tests and how they may improve in the following test. The intensiveness of these peer learning sessions, if well-attended, is likely to develop the resilience required to persist until one succeeds. Support during the engagement phase is essential to improve student retention and success (Tinto & Pusser 2006:1)

#### Transition phase support

This refers to the support that prepares the students for the world of work. According to Tinto and Pusser (2006), during this phase, the person is prepared for interacting in new ways with members of the new group into which membership is sought. Preparing students for transition is a continuous process from the first year as they are socialised into professional values and placed in a range of clinical settings. The final year is a year of consolidating all the learning that has taken place throughout the course of study in a meaningful way that makes sense to the students.

Academic monitoring and support pillars refer to the structures that enable effective implementation of AMS in the undergraduate programme. These include responsiveness, commitment, shared vision, collaboration, relevance and continuity, which have been described earlier in this paper.

Academic monitoring and support architects refer to conditions that may serve as facilitators or threats to effective implementation of AMS. These include political will and support by the leadership of the institution, as the visionary leader who demonstrates commitment to change is invaluable in convincing those with a negative view towards the proposed change (Margerum & Robinson 2015). The availability of resources, students’ engagement with AMS in order to achieve the desired outcomes, strategic collaborative partnerships and implementing the required support in a structured and holistic manner are other important pillars of AMS.

The AMS process and goal-oriented consequences refers to short-term and long-term outcomes of AMS. The short-term outcomes are those experienced during the process of engaging in AMS and the long-term outcomes are associated with the end product of AMS. The short-term consequences include academic resilience and persistence and empowered and confident students. Academic resilience and persistence in this middle-range theory is defined as the student’s increased chance to succeed because of internal motivation to perform better and succeed in the midst of all the adverse factors that place them at risk. Academic resilience is developed through engaging in peer learning activities in the form of supplemental instruction or tutoring by peers outside the curriculum requirements and peer-mentorship in the clinical settings. Academic resilience and persistence is associated with student retention in the programme until they complete the qualification, as stated in Crosling, Thomas and Heagney (2008). An empowered and confident student is defined as a student who is equipped with personal and academic competencies required to cope with the demands of higher education. These are developed through life skills development workshops, participating in peer-mentorship programmes and supported by the academics during the process of engaging in the curricula activities (both formal and hidden curricula).

Goal-related consequences in this middle-range theory include educational equity, improved pass rates and throughputs, change agents, improved distribution of nursing and midwifery workforce and later improved population and health outcomes. Educational equity is defined as a means of achieving equality by providing the best opportunities for all students to achieve their full potential and by acting to address instances of disadvantage, which restrict educational achievement. This definition is in line with UNESCO’s (2015) definition of educational equity. Improved pass rates and throughputs refer to balanced successful completion of qualifications by all the students. This eventually contributes to the available numbers of competent nursing and midwifery staff in the workforce for equitable distribution both in urban and remote areas, resulting in improved service delivery and better population health outcomes. The quality of graduates produced has the potential to serve as a change agent that will contribute to the influencing of change in the healthcare system.

## Limitations of study

In this study, the limitations included inability to interview some of the critical stakeholders and language barriers of key informants.

Inability to interview some of the critical stakeholders: The researcher was unable to interview two key informants because of their busy work schedules. Triangulation of data sources to include document analysis assisted in addressing this limitation. Relevant data were sourced from the reports, minutes of the meetings and other related communication by these key informants.

Language: In this study, some of the cultural informants, although they used English during interviews and natural conversation, also used some isiZulu as it was their first language. The researcher, although she understood basic isiZulu, was unable to freely respond and explore further some of the interesting information shared. In the absence of the research assistant who understood the language, other cultural informants who were part of the conversation assisted with translation for the benefit of all in the mixed interview groups.

## Recommendations

The study revealed unstructured but important peer support in the clinical settings which was part of the comprehensive support. There is a need to develop a structured peer support programme to support new nursing students in the clinical settings, which will be monitored and evaluated for its effectiveness. Further research is recommended to follow up students from remote and rural areas, who are admitted as part of targeted admission and selection policy. This researcher should aim at establishing the support interventions that make a difference in these students’ academic performance as well as their career choices and destination on completion as the emerging evidence shows that students recruited from rural and remote areas, if well supported, are likely to succeed and go back and serve in under-resourced settings.

## Conclusion

The phenomenon of AMS emerged as part of the solution to the high attrition rate of student nurses that later impacts on the nursing and midwifery workforce numbers. The findings revealed that the students who were drawn from diverse backgrounds had access to a range of student support interventions, which were holistic and comprehensive in the form of a student-centred curriculum, academic support offered mainly by peers external to the formalised curriculum, and psychosocial support offered by student support services. The support provided was in phases: pre-enrolment, integration, engagement and transition phases which resulted in students developing self-confidence, acquisition of transferable skills, reduced anxiety and stress levels in clinical areas and improved academic performance.
